# Exploring the utilization of targeted intervention services by transgender individuals in Uttarakhand, India: a qualitative study

**DOI:** 10.3389/fpubh.2024.1476938

**Published:** 2024-12-04

**Authors:** Meenakshi Khapre, Krushna Sahoo, Vartika Saxena, Smita Sinha, Gagandeep Luthra, Anubhuti Joshi

**Affiliations:** ^1^All India Institute of Medical Sciences, Rishikesh, India; ^2^Ministry of Health and Family Welfare, New Delhi, India; ^3^Uttarakhand State AIDS Control Society, Dehradun, India

**Keywords:** transgender, targeted intervention (TI), qualitative study, healthcare utilization model, barriers to service utilization

## Abstract

**Purpose:**

Transgender (TG) women face violence, discrimination, and stigma, which affect their mental health and hinder their access to targeted intervention (TI) services. This lack of access may increase the risk of human immunodeficiency virus/sexually transmitted infections (HIV/STIs). However, the utilization of TI services among transgender women in Uttarakhand, as well as across the country, remains understudied. The purpose of this study is to explore the utilization of TI services by the transgender community in Uttarakhand.

**Methods:**

This qualitative study focused on non-government organizations (NGOs) that implement TI projects in Haridwar and Roorkee, Uttarakhand, India. From September 2023 to January 2024, 5 focus group discussions (FGDs) involving 24 transgender women and 9 in-depth interviews (IDIs) involving NGO staff were conducted. Thematic analysis, guided by the Anderson and Newman healthcare utilization model, was employed.

**Results:**

Several barriers to service utilization were identified, including ritual beliefs, low health literacy, stigma, social isolation, financial insecurity, privacy concerns, and inefficient service delivery. Challenges in the implementation of the STI program and neglect of transgender women in health facilities were also reported. However, enabling factors such as trusted providers, supportive laws, and initiatives by NGOs and government agencies were recognized. Nonetheless, there remains a significant need for information on gender-affirming services and addressing other prevalent health issues within the transgender female community.

**Conclusion:**

The study underscores the interplay of individual, social, and service-related factors affecting healthcare access among transgender women. Inclusive and high-quality healthcare services are necessary to address the complex sociocultural aspects influencing transgender women’s healthcare access and utilization.

## Introduction

Asia has a rich history of gender-nonconforming individuals, often referred to as “transgender.” The *Kama Sutra* describes the sexual lives of these individuals as embodying a “third nature” or Tritiya Prakriti. In India, various transgender identities are recognized, such as Hijras, Aravanis, Kothis, Jogtas/Jogappas, Khusaraa, Pavaiyaa, Chhakka, Kinnar, Zenana, and Shiv-Shakthis (1). Transgender individuals identify with a gender different from the one assigned at birth. They may be categorized as ‘male-to-female’ (MtF), commonly referred to as “transgender female/women,” or “female-to-male,” (FtM), referred to as ‘transgender men.’ (2).

Globally, it is estimated that 2% of people identify as non-binary, transgender, or gender-fluid. In India, approximately 4.8 million transgender individuals were recorded in the 2011 Census (3). However, these figures may not accurately reflect the actual numbers due to challenges in data collection and reporting (4). Transgender women often face stigma and may resort to sex work for financial support, thereby increasing their risk of acquiring sexually transmitted infections (STIs) and human immunodeficiency virus (HIV) (5).

Globally, the standardized HIV incidence rate is 2.56% for transmasculine people and 19.9% for transfeminine people (2, 3). In India, transgender individuals have an estimated HIV prevalence ranging from 3.1 to 9.5%, which is significantly higher than the general population’s HIV prevalence of 0.22% (5, 6). Furthermore, in high-risk sub-populations, the prevalence of treatable STIs such as syphilis, gonorrhea, and chlamydia is significantly higher among transgender individuals compared to others (5).

Transgender women often encounter challenges in accessing HIV care, resulting in adverse health outcomes and increased HIV transmission. Economic exclusion, including a lack of employment opportunities; lower education attainment, including in medical fields; poor housing; and limited access to gender-concordant legal documentation exacerbate health risks (7). Discrimination at health facilities, high medical costs, and inadequate services further hinder access to healthcare, including HIV-related services (8–10).

Research on the healthcare experiences of transgender individuals reveals that clinical services and professionals often fail to understand the specific needs of transgender patients or actively discriminate against them (11, 12).

Transgender women experience higher levels of violence, discrimination, and stigma, which impact their mental health and contribute to substance abuse and suicidal thoughts (8, 9, 13, 14). At the individual level, there is a fear of negative societal repercussions associated with testing positive for HIV (15) These barriers emphasize the urgent need for targeted interventions (TIs) and comprehensive healthcare support for transgender women in India.

HIV prevention and care initiatives that target high-risk groups, such as female sex workers (FSWs), men who have sex with men (MSM), and injecting drug users (IDUs), are known as targeted interventions. Through these measures, people are better equipped to reduce HIV transmission and increase their access to care, support, and treatment resources. The distribution of condoms, syringes, and needles; enhanced access to STI services; behavior change communication through peer counseling; and the creation of an enabling environment by addressing prejudice and stigma are all important components of TIs. In addition, these initiatives encourage at-risk groups to take the lead in community ownership. TIs optimize resources where HIV risk is the highest, rather than replacing more comprehensive community interventions, even if these largely focus on high-risk groups (16). To the best of our knowledge, we could not find any study on the utilization of targeted intervention (TI) services by transgender women. The purpose of this study is was to explore the utilization of TI services by the transgender women in Uttarakhand, guided by the Anderson and Newman healthcare utilization model (17).

The concept comprises three fundamental components that either facilitate or impede individuals’ use of services: predisposing factors, enabling factors, and need-for-care factors. We aimed to determine the needs, obstacles, and specific challenges that transgender women encounter while attempting to access TI services provided by non-governmental organizations under the National AIDS Control Program. These findings will shape targeted interventions aimed at addressing barriers, strengthening facilitators, and ultimately improving health outcomes while reducing HIV transmission among transgender women in India.

## Methods

### Study design, participants, and settings

We employed a phenomenological approach to conduct this qualitative study. The study was conducted within the context of NGOs implementing TI projects registered with the District AIDS Control Society in Haridwar and Roorkee, Uttarakhand, India. These NGOs had already mapped transgender women in their respective areas to provide targeted interventions (TI). Various staff members from NGOs were interviewed, including project managers (PMs), outreach workers (ORWs), counselors, and peer educators (PEs). A representative sample of transgender female adults (>18 yrs) was selected as the study participants, irrespective of their enrollment status with NGOs. Data collection was conducted from September 2023 to January 2024.

All interviews were conducted by trained interviewers experienced in qualitative research. One of the authors had already been working with people living with HIV (PLHIV) cases for the last 3 years and had field experience, as well as proficiency in the local language.

### Data collection methods

The participants were approached through NGO personnel, who were responsible for bringing potential participants along and informing them about the focus group discussion (FGD). Written informed consent was obtained from each participant before the interviews. Five FGDs were conducted at appropriatetimes and locations. The FGD guide explored the participants’ relationship with NGOs, their experiences, and perceived barriers to service utilization (Supplementary material S1). The FGDs were conducted in a quiet room at the NGO office or meeting area, maintaining privacy and confidentiality. Each session lasted approximately 60 min. Demographic and healthcare service utilization data were collected using a structured schedule.

We conducted in-depth interviews (IDIs) with NGO personnel to gain valuable insights into the participants’ perspectives, experiences, and opinions on service utilization by transgender women. With the help of the IDI guide (Supplementary material S2), we discussed the roles and responsibilities of NGO personnel related to transgender individuals, their work experiences, and the barriers and facilitators affecting service uptake by transgender women. Each session lasted for approximately 40–45 min.

### Data management and analysis

We audio-recorded the interviews and discussions while also taking field notes to capture non-verbal cues and group dynamics. At the end of each session, the moderator summarized the meeting and thanked the participants. After a few interviews, we drafted memos to identify the key ideas and emerging themes. The audio recordings were transcribed verbatim the following day, and the transcripts were coded for analysis. Recruitment continued until data saturation was reached, which was determined by the recurrence of similar responses and the absence of new emerging themes.

A thematic analysis using a framework approach was conducted. After familarizing themselves with the transcripts, the authors performed open coding independently, categorizing and identifying the emerging themes. We discussed preliminary themes, patterns, and similarities after conducting the transcript analysis two or three times. We identified a set of codes for the initial analytic framework and used the Anderson healthcare utilization framework model (17). Predisposing factors reflect individuals’ propensity to face barriers when utilizing health services, enabling factors are the resources that may facilitate access to services, and need factors represent the potential identified needs for health service use, such as self-perceived health, severity, and chronic conditions. The framework was later used to structure subsequent analyses. Similar codes were clustered into subcategories and categories (individual, social, and service-related aspects), and underlying themes were identified.

Throughout the process, discussions were conducted regarding the differences in coding and delineating themes. The coding of the data was performed using MAXQDA software (MAXQDA Analytics Pro 2020, VERBI GmbH, Berlin, Germany). The Consolidated Criteria for Reporting Qualitative Research (COREQ) guideline were followed for reporting the study.

### Ethical considerations

Written permission was obtained from the funding source. Institutional ethical approval was granted. We provided detailed information about the study’s purpose, confidentiality, and voluntary participation.

## Results

Two distinct perspectives emerged from these discussions: one from the transgender women’s viewpoint and the other from the providers’ standpoint. These perspectives were systematically organized into themes, categories, and codes using the Anderson framework model (Supplementary Figure S1) to elucidate the barriers and facilitators influencing the utilization of services within the transgender female community (Supplementary Tables S1, S2).

### Recipient’s perspective on barriers and facilitators to the utilization of services

A total of five FGDs were conducted across Haridwar, Roorkee, and Bhagwanpur, involving 24 transgender women representing different religious affiliations. Of the 24 participants, 19 were recruited into the study by the NGOs. The median age of the participants was 25 years, and their educational levels varied significantly, with 12.5% being illiterate to 4.2% holding graduate degrees. The median income was Rs. 8,000 (IQR Rs. 2,000–Rs. 40,000), sourced from activities such as sex work (29.2%), begging (25%), dancing (29.2%), labor (8.3%), private employment (4.2%), and support from family/friends (4.2%). Financially, 29.2% reported having enough money, while 66.7% could barely sustain themselves. Over the past 6 months, 50% rented homes, 9.2% owned houses, and 20.8% lived with friends or family, with 58.3% experiencing homelessness at some point.

Regarding STI services, 50% relied on government doctors, 8.3% on private doctors, and 41.7% on NGOs. Nearly all participants (95.8%) were aware of TI services. All participants utilized the monthly condoms provided by NGOs. All participants were linked to the Integrated Counseling and Testing Center (ICTC) services, with three and two individuals linked to antiretroviral therapy (ART) centers and the Revised National Tuberculosis Control Program (RNTCP), respectively.

#### Predisposing factors

Individual Aspects: The transgender women had low health literacy and adhered to traditional beliefs and practices for curing diseases, such as seeking “Jhaad phuk” from faith healers or quacks. Some were in denial of any sexual activity as they belonged to the Hijra community, whose main source of income is through badhai (singing, dancing, and conferring blessings).

However, the amount earned is managed by the head of the group. Others have to engage in sexual activity secretly for their livelihood. Their introverted personalities, characterized by hesitation and shyness, were also a contributing factor to poor treatment-seeking behavior. There is a fear of reprisal from family members if they contract the disease as they have already witnessed the rejection that occurred when they disclosed their gender identity. They themselves have witnessed the stigma and discrimination faced by PLHIV, so they fear getting tested and identifying themselves with the disease.

Poor mental health, characterized by isolation, depression, and challenges in disclosing their identity, further complicated service utilization. Financial constraints, such as the absence of government IDs, loss of wages, and travel costs, were also reported. A few participants reported risking exposure for an additional Rs 200–300 through unprotected sex.

Social Aspects: Social dynamics included a lack of family support, migration challenges, restrictions in dera settings, and discrimination. To avoid humiliating their families, they work in distant places and continue migrating to remain anonymous. Being in a dera (closed community), they had to strictly follow the norms to ensure the guardianship and protection of the Gurus. There were instances of bullying by the police and school teachers.

*“I was made to hold a box of condoms in my hands, and then they took me to the police station. They told me that I had HIV and then drove away in the car.”* (transgender woman, 24 years old).

Service-related Aspects: Barriers related to service provision included a lack of privacy, poor condom quality, and limited availability of lubricants. They reported instances of condom breakage and, as a result, preferred to buy branded condoms from pharmacies. Services primarily focused on PLHIV, excluding treatment for other ailments.

*“HIV is not the only illness. There are many in the high-risk group, okay. Hepatitis is common these days, okay, and jaundice is also widespread. These should also be focused on.”* (transgender woman, 25 years old).

The participants reported inadequate testing facilities for confirmation and timely viral load monitoring. After the HIV screening test, they had to wait for months for confirmation. The unavailability of regular viral load tests had led to dissatisfaction with the medications. Negative experiences while accessing services at government hospitals, expenditures incurred for STI services, lack of NGO support, inappropriate referrals, and being photographed while receiving services were also identified.

*“I will never go there, not at all. Come here, go from here to there, they throw you from one place to another. It is better to take the money from your pocket and immediately apply the medicine; you are cured, sitting at your home.”* (transgender woman, 26 years old).

#### Enabling factors

Individual Aspects: The enabling factors included awareness of testing and disease, readiness to engage with NGOs, self-realization and acceptance of being transgender, and proximity from residence to service centers. This enabling process begins with self-acceptance and the decision to enroll with an NGO.

Social Aspects: Peer support groups motivate and help other transgender women to enroll with an NGO and access services.

Service-related Aspects: PEs play a key role in building trust with NGOs. Transgender women participate in outreach camps and health education activities organized by NGOs. Regular and free supplies of condoms, testing, counseling services, and vocational support were reported to be key factors in availing services. NGOs as a hub for relaxation and community interaction were also highlighted. NGOs provide linkages to other service providers in case of migration or specific health needs.

#### Need factors

Individual Aspects: The transgender women perceived STIs to be highly prevalent and severe diseases.

*“We fall in a high-risk group; we generally have sex, and if we have sex, then these two things should be treated properly.”* (transgender woman, 32 years old).

The participants expressed the need to couple STI-related health education with other relevant topics, such as hormone replacement therapy (HRT) and gender-affirming surgeries.In the absence of proper knowledge, they end up consuming high doses of contraceptive pills over a long period.

Social Aspects: NGOs should strive to provide social support, including employment opportunities and ration support.

Service-related Aspects: The participants conveyed the need for professional and dedicated doctors in the provision of services.

### Provider’s perspective on barriers and facilitators to the utilization of services

The in-depth interviews included two PMs, two counselors, two ORWs, and three PEs working in NGO’s.

#### Predisposing factors

Individual Aspects: For enrollment in NGO services, 20 years is the minimum age requirement when an individual is already engaging in sexual activities. The majority of transgender women are uneducated and lack awareness or perceived need for health services.

*“They stand at the bus stop, they stand at the railway station, they stand there for the sake of sex, they earn money—that’s all they are concerned with. They are not concerned about medicine or the hospital.”* (Participant no. 6).

Mental health issues, such as depression and substance abuse, along with financial insecurity and migration for work, were found to be the prevalent issues.

Social Aspects: Discrimination against transgender individuals, along with hierarchical and violent environments within the dera system, added to the complexities of service utilization.

Service-related Aspects: Issues related to services, such as the ineffective implementation of STI prevention programs, were highlighted. Concerns included the absence of standardized testing protocols, the shortage of essential supplies such as condoms and testing kits, and the lack of systems to monitor condom usage. Referral services encountered challenges such as out-of-pocket costs, long waiting times, and privacy concerns. Healthcare workers complained about inadequate travel reimbursement for outreach, and anomalies were reported in the selection process for PEs. The providers also mentioned the difficulty of persuading and gaining the trust of transgender women.

#### Enabling factors

Individual Aspects: Rationing during the COVID-19 pandemic influenced service accessibility. They also assist transgender women with legal settlements.

Social Aspects: An NGO was regarded as a place where transgender women could meet and enjoy each other’s company.

Service-related Aspects: The enabling factors included the enforcement of the Transgender Act. NGOs have implemented various strategies, such as hotspot and crisis management committees, in addition to linking beneficiaries to other providers based on their health needs. Some effective strategies include social marketing of condoms, incentivization, liaison with private labs, health education, meetings with transgender women, outreach activities, and reminders for follow-up visits.

The state has also contributed by monitoring the supply chain of commodities, establishing dedicated Outpatient departments (OPD) for transgender women, and providing communication skill training for NGO staff.

#### Need factors

Finally, the providers emphasized the need for free, quality healthcare for transgender women. Additional services, such as Aadhar card provision and hostel facilities, could also attract more transgender women to utilize healthcare services.

*“If someone does not want to stay as a Hijra, then there should be some kind of support so that they can find work, something like this should be done so that they can live somewhere.”* (Participant no 1).

## Discussion

A total of 24 transgender women, aged between 18 and 45 years, participated in the study. Their sources of income included labor, sex work, begging, dancing, and other activities; 66.7% of them reported experiencing financial difficulties. Homelessness (58.3%) increased the complexity of the issue. In the present study, the participants regularly utilized only free condom services, in contrast to a study conducted in Yogyakarta (18), where participants regularly accessed most services.

To the best of our knowledge, this is the first sizeable qualitative study in India that triangulated findings from the perspectives of both the beneficiary and the provider to identify complexities and discrepancies in service provision. Record-based data were also collected, which confirmed the qualitative findings (Supplementary material S3). We used the Anderson framework model, developed from a large quantitative survey, and continuously updated and modified according to specific settings.

From the perspective of the transgender women, ritual beliefs to cure disease and low health literacy emerged as barriers, leading to hesitancy and poor health-seeking behavior. Stigma and fear surrounding STIs, along with social isolation and financial insecurity, contribute to the reluctance of transgender women to engage with TI services. A study conducted in Nepal highlighted significant levels of healthcare-related stigma, anti-trans stigma, and prejudice in society, reflecting the complex sociocultural environment transgender people navigate (19). Studies have shown that transgender individuals avoid discussing their gender identity and sexual health with healthcare providers due to stigma and concerns about confidentiality (20, 21). Previously engaged in traditional occupations such as ‘badhai,’ transgender women now resort to sex work or begging for a livelihood due to educational disruptions, unequal job opportunities, and economic challenges (22). High rates of school dropout, caused by social bullying in educational institutes for not adhering to gender norms, further exacerbate these issues. As a minority, a significant proportion of transgender women also experience poor mental health, including substance abuse (13).

Venkatesan Chakrapani identified a syndemic of psychosocial health conditions among MSM and transgender women in India (9). Hijras were found to have a significantly higher prevalence of HIV as they are more likely to receive money for sex and have an earlier sexual debut (23, 24).

Social aspects compound the barriers as a lack of family support and a need for belongingness lead individuals to attach themselves to groups (dera / gharanas). For transgender individuals living in the dera system, their access to care is dependent on obtaining permission and support from the guru, as reported by Raghuram (10). Transgender women living in the Dera system believe they do not belong to the outside world and deliberately communicate in a different language, “Farsi,” so that it is difficult for other people to understand when they communicate. Their health-seeking behavior was also understood to be very low. They are only allowed to leave the Dera with permission from their teachers,and they earn their wages by dancing on various occasions. They do not engage in any sexual activity inside the Deras. A portion of the money earned from dancing is allocated to their respective teachers. Approximately 40–50% of the transgender women reported being discriminated against and bullied by the police/local leaders (25). Within the domain of health service provision, inadequate privacy, questionable condom quality, insufficient testing facilities, uncomfortable service experiences, and a lack of hormone replacement therapies contribute to the perceived barriers. Thakur et al. found that participants who received condoms from peer educators were 1.74 times more likely to use condoms during their last sexual intercourse (26). Transgender women mostly have sex with men, usually forceful rough/anal sex. Therefore, the inspection of standard-quality condoms provided free of charge revealed instances of condom breakage, which affected trust in the service (27).

The participants raised concerns about being photographed while receiving services, which affected their privacy. Surprisingly, we found that many transgender women were abusing hormonal pills without any knowledge of the side effects. They feel trusted and connected when counseled and managed by someone who can closely relate to them. However, the providers expressed concerns regarding the discretionary peer selection process. Scheim reported that trusted providers and integration with hormone therapy monitoring could facilitate the provision of services (28). Although the third gender is now included in the Aadhar card (the unique identity number issued by the Government of India), which is required to avail government schemes, many were unaware, and the NGO services were mainly limited to HIV and STDs. This led to significant out-of-pocket expenditures for chronic and common illnesses, as reported by Gupta et al. (29).

From the provider’s viewpoint, pervasive issues included suboptimal implementation of STI programs, deficiencies in testing procedures, and scarcity of essential consumables and testing kits. Transgender women feel excluded in health facilities designed around the binary of male and female and are often neglected, as also reported by Raghuram and Gupta (10, 29). Itogenesis theory primarily focuses on how doctors create boundaries while treating transgender women and the discrimination they face during care (30).

On the positive side, the enabling factors included the enforcement of the Transgender Act 2019, the HIV Act 2017, and commendable efforts by NGOs and the state government. The majority of transgender women reported visiting NGO offices to bond and express themselves freely. Accessibility and peer support were other facilitators for utilizing health services.

The present study showed that the transgender women were well aware of their STD risk and the severity of the disease. Many projects, such as Avhaan, Pehchan, and Yogyakarta (18, 31, 32), can be considered models. There is a need to focus on broader, more inclusive health services that encompass employment opportunities, social security schemes, and gender-affirming health services.

The perspectives of the transgender women and providers were almost aligned with each other. The minimum age requirement for enrolling in an NGO’s service is 20 years, but many transgender women engage in sexual activity much earlier. Fontenot commented that transgender youth face barriers to self-efficacy in sexual decision-making and safety concerns in relationships (33). While the providers confirmed the free availability of condoms, the transgender women reported being dissatisfied with the quality of condoms and the unavailability of lubricants. Social support and stigma played crucial roles in the transgender women’s experiences. This study highlights the importance of addressing broader socio-cultural factors influencing healthcare access for marginalized populations, emphasizing service availability and positive social interactions.

### Limitations

The study has some limitations. The findings may not be generalizable to other parts of India due to cultural complexities. The participants might not have disclosed very sensitive information.

### Implications for policy and practice

The study highlights the need for creating supportive educational environments and job opportunities. It is crucial to simplify the transgender certificate process, which currently hinges on medical procedures. It is also essential to address specific needs, and provide quality condoms, lubricants, and gender-affirming care. In addition, services should go beyond STIs and include livelihood support to break financial dependence and reduce risky behaviors. Finally, it is important to enroll transgender individuals in health insurance schemes such as Ayushman Bharat for broader healthcare access.

## Conclusion

Individual, social, and service-related issues were interwoven through themes such as predisposing, enabling, and need factors. The predisposing factors included low health literacy, stigma, poor mental health, financial insecurity, poor service availability (essential test kits, condoms, and HRT), accessibility issues (lack of hospital networking, minimum age for enrollment), and acceptability concerns (binary gender in the medical system). The enabling factors included awareness through NGO campaigns, integrated services (ART, the ICTC, and private laboratories), trustworthy providers (PE), and the existence of HIV and transgender laws. Perceived disease prevalence and severity, the need for belongingness, and respectful, quality, need-based health services were identified as the need factors for availing services. It is recommended to conduct a thorough investigation into the iatrogenic discrimination faced by transgender women when they seek healthcare services, as well as to understand the perspectives of healthcare professionals on this issue. This will help in identifying and addressing the barriers to equitable healthcare for transgender women.

## Data Availability

The original contributions presented in the study are included in the article/Supplementary material, further inquiries can be directed to the corresponding author/s.
