# Supplementary material for: Exploring the utilization of targeted intervention services by transgender individuals in Uttarakhand, India: a qualitative study
Source: Front Public Health. 2024 Dec 4;12:1476938. doi: 10.3389/fpubh.2024.1476938 (PMC11652492; doi:10.3389/fpubh.2024.1476938)
Supplement: Supplementary file 1 [file Data_Sheet_1.PDF]

<u>Supplementary appendix 1 (SA1)</u>

Focus Group Guide

Study Title: Barriers in service uptake for TG/TS in TI program

Focus group number:

Date:

Facilitator Initials:

(NOTE TO FACILITATOR: Read only bolded text. Additional text is provided to the prompt. We have ideas for probes, so read-only as needed to guide the conversation.)

Recent studies have found that Transgender people Often have difficulty accessing HIV care, which can lead to poor health outcomes and increased HIV transmission. The purpose of this focus group is to find out barriers that may be unique to transgenders and elucidate the observed disparities in uptake of TI services.

By offering verbal consent, you have agreed to participate in a focus group to discuss this topic. We thank you in advance for your participation. Your thoughts are important for us to understand the barrier for transgender.

Discussion format:

We would like the discussion to be informal, so there’s no need to wait for us to call on you to respond. In fact, we encourage you to respond directly to the comments other people make. Please speak one at a time so we can all hear what is being said. If you don’t understand a question, please let us know. We are here to ask questions, listen and make sure everyone has a chance to share. There are no wrong or right answers.

As you saw in the consent, we will be audio recording the discussion, because we don’t want to miss any of your comments. No one outside of this room will have access to these recordings and they will be destroyed after our report is written.

Introductions:

(Introduce yourself and your co-facilitator.) I’m going to ask each of you to introduce yourself to the group. Please only give a first name. Also, the name you choose to introduce yourself does not need to be the name you usually use. You can introduce yourself using any first name you choose. (Have participants introduce themselves by their first name only.)

Ground rules:

Now I want to go over a few guidelines for the group discussion.

1. What we say in the group should stay here – keep it confidential.

2. One person talks at a time. Because we would like to hear from everyone, I might call on you if you're not saying much, or ask you to give others a chance if you have talked a lot.

3. Feel free to respond to another group member, not just to my questions. You can follow up on what someone has said, agree or disagree, or give an example. Please speak for yourself using "I" statements and avoid attacking or putting someone else's ideas down.

4. We'll be talking about TI services and barriers, which can be a sensitive topic. It's OK to talk about anything at all here. We really want to hear from you. If you prefer, you may talk about transgender in general instead of your own experiences if you would like.

5. We want to hear a lot of different perspectives, so as a group we have to respect everyone's point of view. Everyone won't agree, but we want to hear from everybody. There are no wrong answers! We want to ensure a safe environment where individuals speak openly so remember that everyone's opinion and experience is valuable.

6. To protect everyone's privacy, please use only the first names for everyone here.

7. Feel free to get up from the table at any time. You may want to get more snacks, go to the restroom or just take a break or a stretch.

8. Please take a moment now to turn off all cell phones or place them on silent. (Give them a moment now to do this.)

Are there any ground rules that we should add to these? (Give them a moment to discuss.)

Can everybody work with these guidelines? (Allow them to confirm.)

Okay, let's begin. I will turn on the digital recorders now.

(TURN ON DIGITAL RECORDERS.)

Introductions/ Ice Breaker:

I'm going to ask each of you to introduce yourself to the group. Please only give a first name. Also, the name you choose to introduce yourself does not need to be the name you usually use. You can introduce yourself using any first name you choose. Please also let us know where your name came from and what special meaning it has.

<u>TI services knowledge</u>

1. To begin our conversation, what have you heard about TI (name of NGO) and its services? **(If participants are unable to understand the term, then explain TI services)**

- (Behaviour Change Communication including peer educator
- Condom Programming
- STI Care and Counseling
- Creating an enabling environment: Through addressing the macro environment in which risk behavior takes place and facilitating legal and policy changes that will facilitate preventive behavior.)

Potential probes:

- Are people you know talking about TI? If so, what are they saying? (assess their knowledge of TI services)
- Have you or known anyone who has taken any TI services or service providers? What was your / their experience?
- In details like what had happened, who had helped them who had not, not motivated to use services, understanding about the risk of disease

2. Let's talk a little more about the facts about TI. Can anyone be willing to share their thoughts and knowledge? (Pause)

Probe: **Encourage participants to share their understanding of TI services and their role in HIV prevention.**

Targeted interventions are aimed at offering prevention and care services to high-risk populations (Female Sex Workers- FSW, Males having Sex with Male- MSM and Injecting Drug Users- IDUs) within communities by providing them with the information, means, and skills they need to minimize HIV transmission and improving their access to care, support and treatment services.

These programs also improve sexual and reproductive health (SRH) among these populations and improve general health by helping them reduce the harm associated with behavior such as sex work and injecting drug use.

3. What are the initial things that come up for you when you think about using TI services as and when required?

(What are the thoughts that came into your mind before seeking TI services)

(Attitude of Transpeople while availing services)

Potential Probes

- What is the perceived need for yourself and others?
- Can you share any previous experiences with TI services?
- When seeking care, what aspects come to mind, such as appointment availability, health worker attitudes, concerns about care quality, drug/condom availability, costs, travel distances, or lack of knowledge about when and where to seek care?
- Are there any social issues, like concerns about what friends or family might think, potential effects on employment if others find out, or stigma associated with seeking care?

4. How comfortable would you and/or your trans friends feel discussing the RTI/ STI symptoms with medical providers in TI clinics?

Probe:

- *Do you believe that transgender individuals in your community are generally comfortable in TI clinics? Why or why not?*
- *If not, where do they seek treatment and why? How much does it cost them?*

(Explain STI/RTI symptoms if they are not aware, Ask- have you ever experienced STI/RTI symptoms)

5. Would you and your transgender friends be willing to get tested for HIV once a year? Why or why not?

- ***Probe:*** How often do you and your transgender friends typically get tested for HIV? Where do they go for testing, and are there cost considerations

6. Do you think that you and your transgender friends would be willing to use condoms consistently?

- ***Probe:*** How often do you and your transgender friends use condoms from TI services? If not regularly from these services, where do they obtain them, and why? Are there cost considerations?

7. Would you / your trans friend be willing to attend health education sessions on Sexually transmitted diseases and ways of prevention?

Probe: When was the last time you attended such a session, and who organized it? Can you describe any benefits you received from attending?

(Can you share your experiences regarding the quality of care and support you have received from TI personnel when accessing TI services?

Probe: What aspects of care were particularly positive or negative in your experience?

Probe: Have you encountered TI personnel who provided gender-affirming care? How did that make a difference in your experience?)

Concluding thoughts

How can govt improve on TI services?

- Removing stigma, improving appointments, training of personnel, more IEC activities, some insurances, etc.

Thank you very much for your time today. I would like to remind everyone that we have asked for everything that has been said today to be kept confidential. The information you have provided will be very helpful in helping us to think about improving the TI services for transgenders.

The main challenges or difficulties you and your transgender peers face in accessing and utilizing TI prevention and care services?

- provide specific examples of these challenges or difficulties

Describe the level of awareness about TI services among transgender individuals.

- Are there any misconceptions or myths about TI services
- Have you observed any efforts to increase awareness about TI services in your community?

What role does stigma or discrimination play in preventing transgender individuals from accessing TI services?

Are there any specific concerns or fears that deter you or your transgender friends from using TI services?

- Can you elaborate on these concerns or fears and their origins?
