# Supplementary material for: Exploring the utilization of targeted intervention services by transgender individuals in Uttarakhand, India: a qualitative study
Source: Front Public Health. 2024 Dec 4;12:1476938. doi: 10.3389/fpubh.2024.1476938 (PMC11652492; doi:10.3389/fpubh.2024.1476938)
Supplement: Supplementary file 2 [file Data_Sheet_2.PDF]

Supplementary appendix 2 (SA2)

Indepth interview Guide

Study Title: Barriers in service uptake for TG/TS in TI program

IDI number:

Age :   Gender ________________

Affiliation :   ORW/ PE / Project manager / Counsellor/ Doctor / Community mobiliser /Specify ________________

Years of experience   Date:

Facilitator Initials:

(NOTE TO FACILITATOR: Read only bolded text. Additional text is provided to prompt. We ideas for probes, so read only as needed to guide the conversation.)

Recent studies have found that Transgender people Often have difficulty accessing HIV care ,which can lead to poor health outcomes and increased HIV transmission. The purpose of this focus group is to find out barriers that may be unique to transgenders in uptake of TI services.
By offering verbal consent, you have agreed to participate in a focus group to discuss this topic. We thank you in advance for your participation. Your thoughts are important for us to understand the barrier for transgender women.

Discussion format:

We would like the discussion to be informal, so there's no need to wait for us to call on you to respond. In fact, we encourage you to respond directly to the comments other people make. Please speak one at a time so we can all hear what is being said. If you don't understand a question, please let us know. We are here to ask questions, listen and make sure you has a chance to share. This will take 40-50 minutes. There are no wrong or right answers.
As you saw in the consent, we will be audio recording the discussion, because we don't want to miss any of your comments. No one outside of this room will have access to these recordings and they will be destroyed after our report is written.

Introductions:

(*Introduce yourself and your co-facilitator.)*

Ground rules:

Now I want to go over a few guidelines for the group discussion.

1. **What you say should stay here – keep it confidential.**
2. **Feel free to respond.**
3. **We'll be talking some about TI services and barriers, which can be a sensitive topic. We really want to hear from you**
4. **Just tell your experiences and perception , there is nothing right or wrong here**

1. **What is your roles and responsibilities in project especially related to transgender ?**

   Probe : defined roles, not clearly defined roles, conflicting roles , if there is clarity in roles,

2. **To what extent do you think you were able fulfil your roles and responsibilities related to transgender ?**

   Probe – instance when you could fulfil the role and when not. What were the reason for same

3. **How willing are transgender to take up the TI services**

   Probe – which services they take up most and why. Which they take up least and why.

4. **Tell us in more details about the barriers you perceive while rendering TI services to transgenders?**

- Manpower – staff turnover , less staff
- Time
- Money
- Material (condom, STI treatment kit)
- Training related
- Communication
- Community hesitation etc
