# Supplementary material for: Exploring the utilization of targeted intervention services by transgender individuals in Uttarakhand, India: a qualitative study
Source: Front Public Health. 2024 Dec 4;12:1476938. doi: 10.3389/fpubh.2024.1476938 (PMC11652492; doi:10.3389/fpubh.2024.1476938)
Supplement: Supplementary file 3 [file Data_Sheet_3.PDF]

<u>**Supplementary Tables- S1,S2**</u>

Supplementary Table S1- Utilization of Targeted intervention services among transgender: Provider's aspect

**Theme**	**categories**	**subcategories**	**Codes**
**Predisposing**	Individual factor	Minimum age of receiving service	After 20 recruitment
		Uneducated and unaware	Lack of concern
			No felt need
		Fear of disease and death	Fear of meeting family with HIV
			Denial of disease, sexual activity
			Non-disclosure
		Perceived Stigma	
		Negative experiences with health service	Indifference towards govt services
		Poor mental Health	Depressed and substance abuse
		Financial insecurity	Demanding hard work for a livelihood<br>Migration
	Social factor	Discrimination	
		Dera (community group) system	Hierarchy and violent environment
	Service-related factor	Poor implementation of STI	Non-standardized test
			Low availability consumables stock (condom, jelly, testing kits for CD4 , STDs)
			Non-tracking of actual condom usage
		Poor Referral services	Out-of-pocket payments
			Long waiting time
			Lack of privacy
		Health worker related	Insufficient TA for outreach
			Irregularity in PE selection

			Unable to build trust in the community
**Enabling**	Individual factor	Provision of social security	Ration during Covid times<br>Legal settlements
	Social factor	Sense of belongingness	Forum to express gender identity
		Availability of condom	
	Service-related factor	Enforced TG act	
		Efforts made by NGO	Hot spot and crisis management committee
			Linking with NGO
			Social marketing branded condoms
			Incentivize for work
			Liaison with PP and lab
			Health education and meetings with TG
			outreach activity
			repeated visit reminders
		Efforts made by the state	Monitoring the Supply chain of commodities
			Dedicated OPD
			Skill training of NGO staff
**Need**	Individual factor	Inability to negotiate sex	Unprotected sex for extra money
			Easy money and unemployment
			Anal sex
	Social factor	Social support	Other services like Aadhar card, hostel, etc.
	Service-related factor	Dedicated professional doctors	Free, quality, respectful and privacy during service uptake

Abbreviations-

HIV: Human Immunodeficiency Virus

CD4: Cluster of Differentiation 4

STDs: Sexually Transmitted Diseases

TA: Travelling Allowance

PE: Peer Educator

NGO: Non-Governmental Organization

PP: Private Practitioner

TG: Transgender

OPD: Out-Patient Department

Supplementary Table S2- Utilization of Targeted intervention services among transgender: Transgender's aspect.

Theme	Categories	Sub categories	Codes
**Predisposing**	Individual Factor	Health belief and attitude	Trust alternative medicine
			Denial of disease
		Introvert personality	Hesitation and shyness
		low health literacy and awareness	Educational sessions, no felt need, peer educator , poor treatment seeking, trust ritual belief and practices to cure disease
		Stigma and fear	STI not revealed
			Fear of reprisal by family, Fear related to disease, denial of disease, hesitation to avail services
		Poor mental health	Isolated, depressed
			Disclosure of identity
		financial insecurity	No aadhar card with TG
			Loss of wages, travel cost
			Risking exposure for more Earning
			Migration
	Social factor	Need of belongingness	Lack of family support, Restriction in dera
		Discrimination	Bullied by police and other
	Service related factor	Lack of privacy	Uncomfortable being photographed
		low condom quality and availability of lubricant	
		Services Limited to PLHIV	Regular blood testing only for PLHIV
			No treatment for other ailments
		Lack of testing facility	Confirmability test
			Timely viral load
		Negative experiences in health facility	Ill treatment, unattended, ignored, long waiting , Lack of facility, out of pocket expenditure, Referred to

			other facility , less support from NGO
**Enabling**	Individual factor	Awareness of testing and disease	HIV mode of transmission and t/t, use of condom, readiness to use service
		self-realization and acceptance	Being Trans
		Proximity of residence	Residing nearby
	Social factor	Supported of the same group members	
	Service-related factor	Trust and feel supported by NGO worker	Speak openly, respected, Hub to relax and meet with group
		Outreach Camps and health education activities	
		Free and regular supply of condom	
		Peer educator from same group	
		Health checkup, testing and counselling services	Availability of STI kits, check up every 3 monthly
		Linkage with other service providers	ART, ICTC, another NGO etc
		Vocational help	
**Need**	Individual factor	Perceived prevalence and severity of disease	STI prevalent
		Awareness	Education on relevant topic like hormone replacement therapy, sex-affirming surgeries, NCD etc
	Social factor	Support	employment, HRT, ration
	Service-related factor	Professional and dedicated doctor	Aware of gender issues and diseases

Abbreviations-

STI: Sexually Transmitted Infections

TG: Transgender

PLHIV: People Living with HIV

NGO: Non-Governmental Organization

t/t: Treatment

ART: Antiretroviral Therapy

ICTC: Integrated Counseling and Testing Center

NCD: Non-Communicable Disease

HRT: Hormone Replacement Therapy
