# Supplementary material for: Exploring the utilization of targeted intervention services by transgender individuals in Uttarakhand, India: a qualitative study
Source: Front Public Health. 2024 Dec 4;12:1476938. doi: 10.3389/fpubh.2024.1476938 (PMC11652492; doi:10.3389/fpubh.2024.1476938)
Supplement: Supplementary file 4 [file Data_Sheet_4.PDF]

Supplementary appendix 3 (SA3)

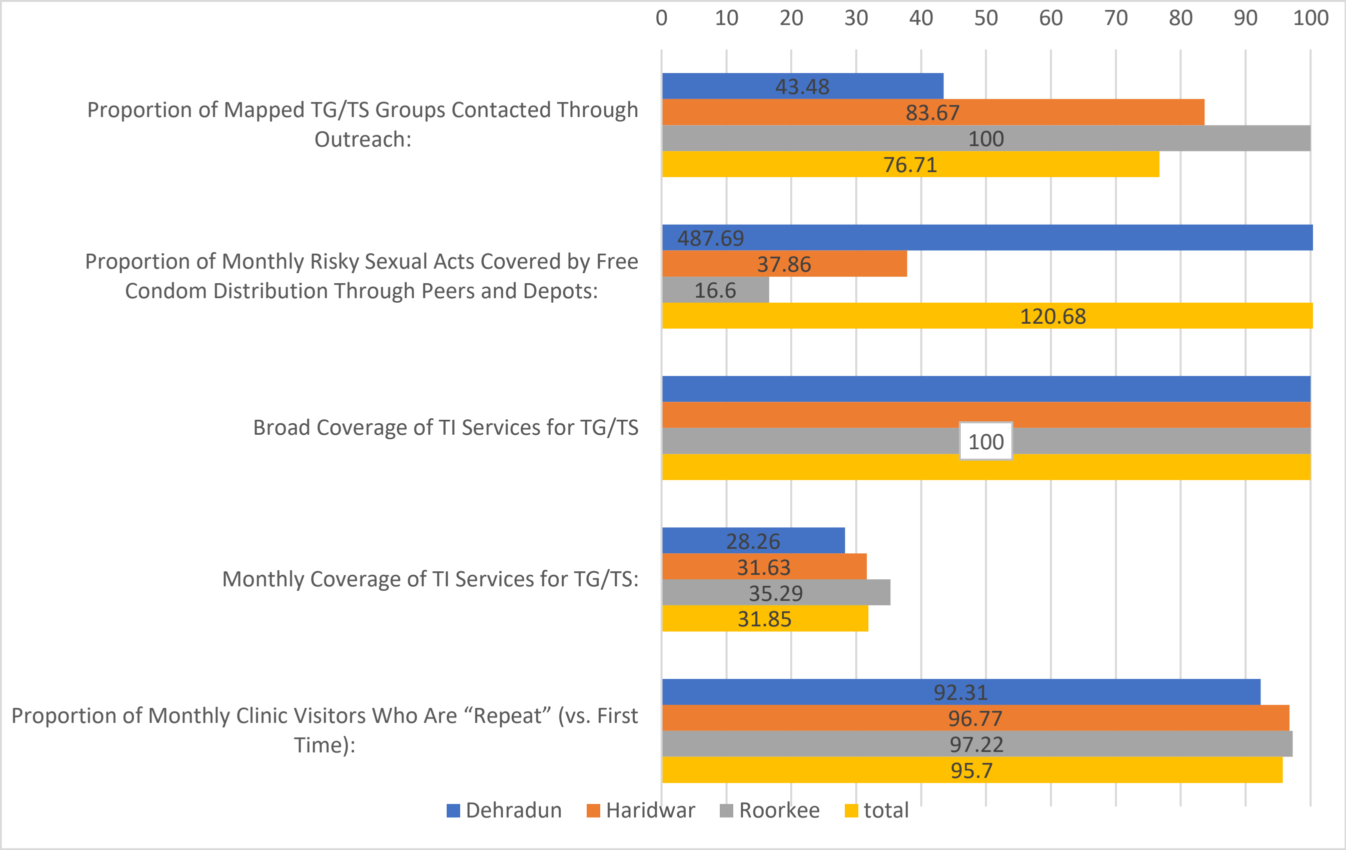

Fig 1 A : Monitoring **Indicators for TI services**

The secondary data on TI service uptake barriers for transgender/transsexual individuals reveals location-specific variations. While contact rates through outreach vary (43.48% in Dehradun, 83.67% in Haridwar, 100% in Roorkee), the coverage of monthly risky sexual acts through condom distribution shows significant differences (487.69% in Dehradun, 37.86% in Haridwar, 16.60% in Roorkee). TI services demonstrate broad coverage (100% in all locations), and monthly service coverage for TG/TS individuals ranges from 28.26% (Dehradun) to 35.29% (Roorkee).

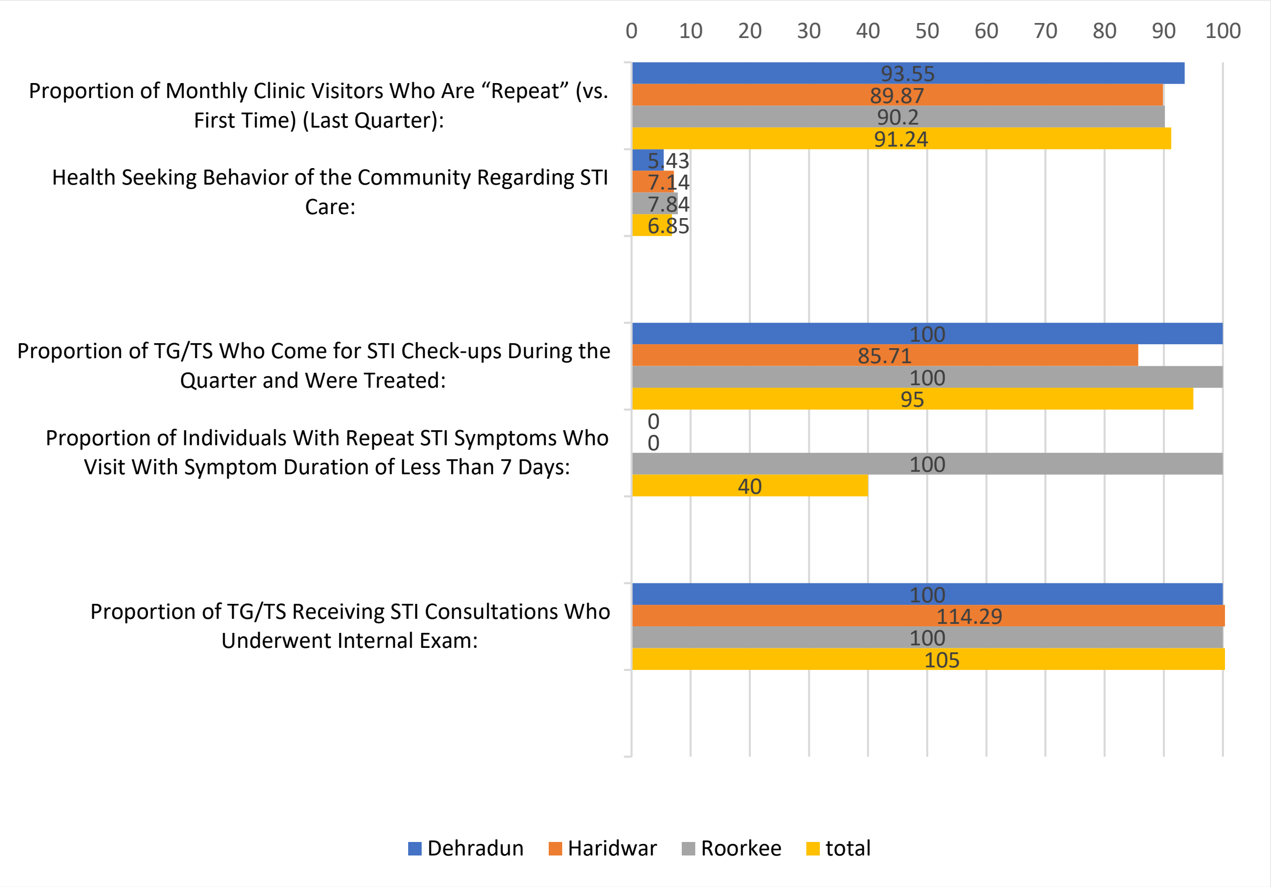

Fig 1 B : Monitoring **Indicators for TI services**

Proportion of monthly and last quarter Repeat clinic visitor were more than 90 % in all centres. Health-seeking behavior for STI care was 5.43% in Dehradun, 7.14% in Haridwar, 7.84% in Roorkee. Proportions of TG/TS individuals seeking STI check-ups and receiving treatment was 85.72% in Haridwar to 100% in Dehradun and Roorkee. Notably, the proportion of individuals with repeat STI symptoms seeking consultation within 7 days ranges widely (0.00% in Dehradun and Haridwar to 100% in Roorkee). Internal exams during STI consultations was 100% in Dehradun and Roorkee, 114 % in Haridwar (extra was due to new registration).
