# Supplementary figures and images for: Exploring the utilization of targeted intervention services by transgender individuals in Uttarakhand, India: a qualitative study
Source: Front Public Health. 2024 Dec 4;12:1476938. doi: 10.3389/fpubh.2024.1476938 (PMC11652492; doi:10.3389/fpubh.2024.1476938)

## Supplementary Figure ( SF1 )

Fig 1 - Anderson Framework model

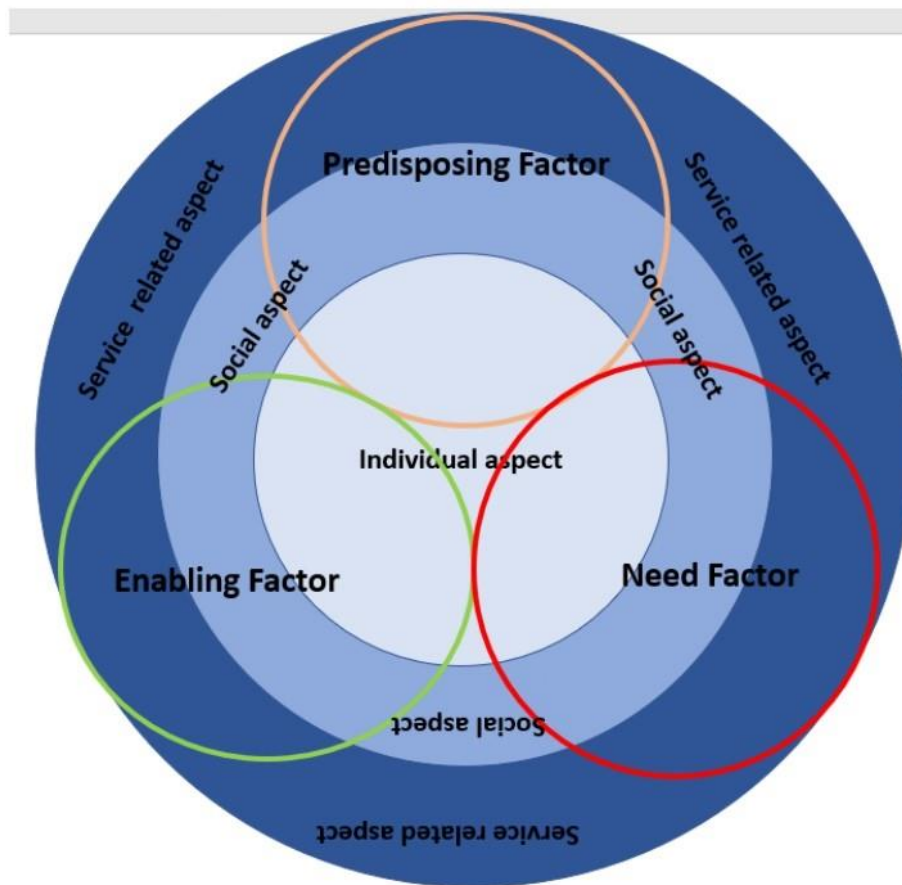

Supplement: Supplementary file 5 [file Data_Sheet_5.PDF]
